# Molecular Basis of Bacterial Host Interactions by Gram-Positive Targeting Bacteriophages

**DOI:** 10.3390/v10080397

**Published:** 2018-07-28

**Authors:** Matthew Dunne, Mario Hupfeld, Jochen Klumpp, Martin J. Loessner

**Affiliations:** Institute of Food Nutrition and Health, ETH Zurich, Schmelzbergstrasse 7, 8092 Zurich, Switzerland; hupfeldm@protonmail.ch (M.H.); jochen.klumpp@hest.ethz.ch (J.K.); martin.loessner@ethz.ch (M.J.L.)

**Keywords:** gram-positive bacteria, bacteriophage, infection, receptor-binding proteins, phage technology, *Listeria monocytogenes*, *Lactococcus lactis*, *Bacillus subtilis*, *Staphylococcus aureus*

## Abstract

The inherent ability of bacteriophages (phages) to infect specific bacterial hosts makes them ideal candidates to develop into antimicrobial agents for pathogen-specific remediation in food processing, biotechnology, and medicine (e.g., phage therapy). Conversely, phage contaminations of fermentation processes are a major concern to dairy and bioprocessing industries. The first stage of any successful phage infection is adsorption to a bacterial host cell, mediated by receptor-binding proteins (RBPs). As the first point of contact, the binding specificity of phage RBPs is the primary determinant of bacterial host range, and thus defines the remediative potential of a phage for a given bacterium. Co-evolution of RBPs and their bacterial receptors has forced endless adaptation cycles of phage-host interactions, which in turn has created a diverse array of phage adsorption mechanisms utilizing an assortment of RBPs. Over the last decade, these intricate mechanisms have been studied intensely using electron microscopy and X-ray crystallography, providing atomic-level details of this fundamental stage in the phage infection cycle. This review summarizes current knowledge surrounding the molecular basis of host interaction for various socioeconomically important Gram-positive targeting phage RBPs to their protein- and saccharide-based receptors. Special attention is paid to the abundant and best-characterized *Siphoviridae* family of tailed phages. Unravelling these complex phage-host dynamics is essential to harness the full potential of phage-based technologies, or for generating novel strategies to combat industrial phage contaminations.

## 1. Introduction

**Figure 1:**
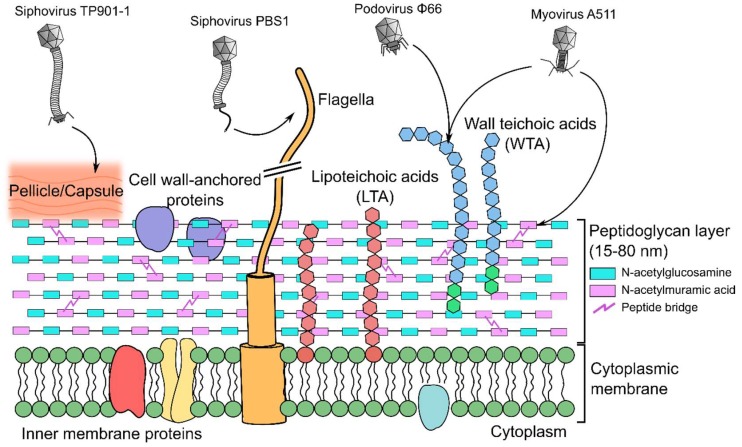
Host cell receptors of Gram-positive targeting phages. Surrounding the cytoplasmic membrane is a complex arrangement of different biopolymers that are receptors for different phages: peptidoglycan (PG) (described in Section 8), teichoic acids (Section 9, Section 10, Section 11 and Section 12), polysaccharides such as a pellicle layer (Section 13 and Section 14), and protruding organelles such as the flagella (Section 15). Highlighted are known host cell receptors for four different *Caudovirales* phages described within this review: *Siphoviridae L. lactis* phage TP901-1 [22] and *B. subtilis* phage PBS1 [23], *Podoviridae S. aureus* phage ϕ66 [24], and *Myoviridae Listeria* phage A511 [20].

Bacteriophages (phages) are viruses that specifically infect bacteria. As the most abundant and ubiquitous biological entities on Earth, with an estimated global population of >10^31^ particles [1], phages play important roles in global ecology and bacterial pathogenicity [2,3]. The first stage of any successful phage infection is adsorption to a suitable host cell. Typically, this involves an initial, reversible, interaction with host cell receptors, before irreversible binding through tighter binding to the initial receptors or through interaction with secondary receptors [4]. Successful host adsorption is a prerequisite to cell wall degradation, penetration, and subsequent DNA transfer into the bacterial host. Receptor binding proteins (RBPs), usually located on the distal end of the phage tail apparatus, mediate binding to the host cell. These proteins of varying complexity and composition interact with a variety of bacterial host receptors, proteins, saccharides, and organelles [5,6] (Figure 1).

Over the last decade, X-ray crystallography and electron microscopy (EM) studies have illustrated a number of phage-host adsorption processes with extraordinary levels of atomic detail [7]. For instance, Taylor et al. used cryo-EM to determine the multifaceted *E. coli* phage T4 baseplate in pre- and post-host attachment states, revealing atomic resolution details of conformation changes to the baseplate prior to DNA ejection [8] that appear conserved in other contractile injection systems, e.g., R-type pyocins [9] and the bacterial type VI secretion system (T6SS) [10]. Furthermore, Hu et al. used cryo-EM to reveal the phage T4 tail tube penetration of the bacterial periplasm that induces curvature of the inner cytoplasm prior to DNA ejection [11]. *Bacillus subtilis* phage SPP1 [12,13,14,15], and *Lactococcal* phages p2 [16,17] and TP901-1 [18,19], among others [20,21] described in this review, have become model systems for understanding the adsorption processes of phages that target Gram-positive bacteria.

The natural ability of phages to infect (and kill) a certain bacterial host range has led to their exploitation over the past decades for the development of diagnostic tools and antibacterials for use in medicine [25,26], food production [27,28,29,30,31], and biotechnology [32,33,34]. For example, phages can be used to treat *Campylobacter* [35] or *Salmonella* [27] infections of chicken flocks, or added to feed to kill *Clostridium* and intestinal coliforms in pigs [36]; added to ready-to-eat foods to prevent *Salmonella* [37] or *Listeria* [38] contaminations; or mixed with various food products to act as bio-preservatives [39,40,41]. Phages and recombinant RBPs have been engineered into affinity molecules for rapid biosensor-based detection of *Staphylococcus aureus* [42,43], *Salmonella* [44,45], *Campylobacter jejuni* [35], or *Shigella flexneri* [46], or attached to magnetic beads for rapid immobilization, magnetic separation, and detection of *Salmonella* [30]. However, virulent phages also present significant challenges to any biotechnological process that is reliant on bacteria, e.g., recombinant therapeutics in *E. coli* [47], milk fermentation to produce cheese and yogurt [48], and many more. Phage infection of lactic acid bacteria (LAB) starter cultures remains the biggest threat for dairy fermentation, causing inadequate milk acidification, poor quality products, and significant economic losses. Addressing this problem is a significant challenge due to the ubiquitous presence of LAB-infecting phages in the environment. *Lactococcus lactis* is the most important bacterial species for the production of cheese, and, consequently, its phages have been intensively studied worldwide, mainly with the aim of developing phage-resistant dairy starter culture strains.

Understanding how phages targeting Gram-positive bacteria recognize their host and absorb to it, i.e., how their RBPs bind specific cell wall receptors, is crucial for understanding phage host range, for the development of novel detection and biocontrol tools, and to evaluate the efficacy of anti-phage mechanisms in fermented food production. This review aims to draw attention to a number of remarkable studies that describe the molecular basis of host recognition by phages infecting Gram-positive bacteria and the influence of their various host cell receptors.

## 2. The Conserved Tail Morphogenesis Sub-Module of Gram-Positive-Targeting *Siphoviridae*

**Figure 2:**
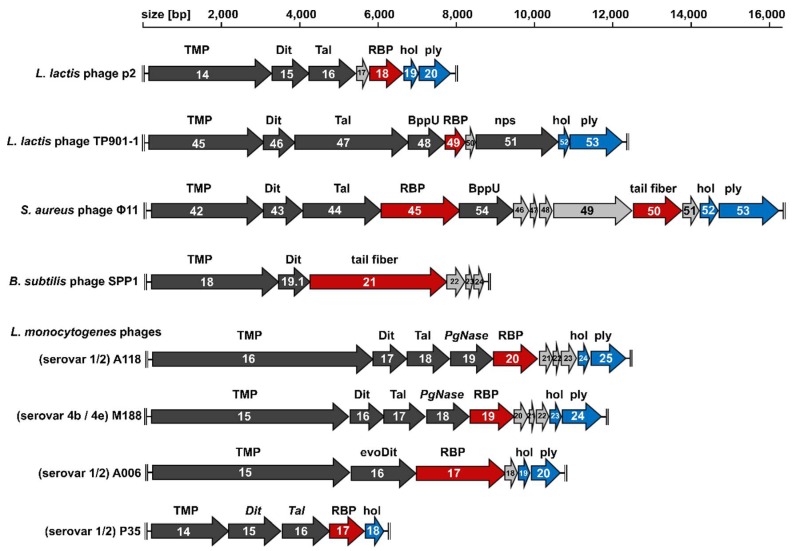
Schematic representation and assignment of the tail morphology genome sub-modules of Gram-positive targeting *Siphoviridae* discussed in this review. Light grey arrows indicate open reading frames (ORFs) encoding hypothetical proteins, dark grey arrows indicate ORFs with known or predicted functions, red arrows indicate the receptor binding protein (RBP) or tail fiber genes, and blue arrows indicate holin and endolysin genes. Abbreviations: TMP, tail tape measure protein; Dit, distal tail protein; evoDit, “evolved” Dit; Tal, tail-associated lysin; BppU, baseplate upper protein; nps, neck passage structure; PgNase, putative peptidoglycan hydrolase; hol, holin; ply, endolysin. Scale bars mark genome positions at 2000-bp intervals.

Most phages described to date belong to the *Caudovirales* order, possessing a double-stranded DNA (dsDNA) genome packaged within a proteinaceous capsid connected to a tail [49]. Based on tail morphology, the *Caudovirales* order is divided into three families: *Myoviridae*, possessing contractile tails; *Podoviridae*, with short stub-like tails; and the most abundant *Siphoviridae*, featuring long non-contractile tails. The majority of characterized Gram-positive targeting phages are *Siphoviridae* [6], and as such, the adsorption mechanisms used by their RBPs is the major focus of this review. Typically, phages feature a modular genome organization, where structural proteins are encoded in the late gene region in a conserved, successive order of their assembly process [50]. In Figure 2, the tail morphogenesis sub-modules—between the tail tape measure protein (TMP) and the lysis cassette (holin and endolysin)—of different Gram-positive targeting *Siphoviridae* are aligned to highlight the consistencies and differences between their baseplate components. Below, we describe the major components of the sub-module that are involved in host adsorption and describe how they produce a diverse assortment of host adsorption mechanisms.

## 3. Distal Tail Protein (Dit)

**Figure 3:**
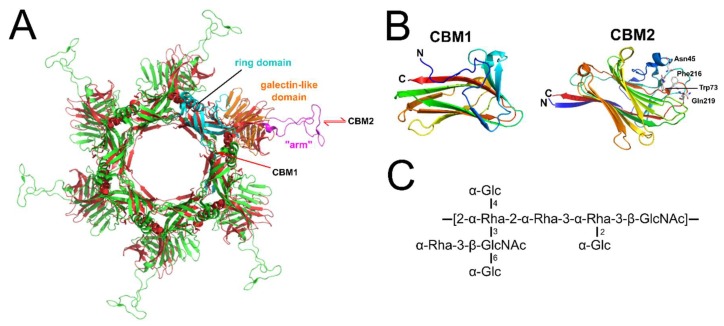
“Evolved” Dits feature with receptor-binding carbohydrate-binding modules (CBMs). (**A**) Cartoon superposition of the hexameric Dit hub structures from *L. lactis* phage p2 (gp15; green) [16] and *B. subtilis* phage SPP1 (gp19.1; red) [12] shown passing through the central symmetry axis of the tail. The domains of a single p2 Dit protein are colored separately: cyan, N-terminal ring domain (residues 1 to 136); orange, galectin-like domain (residues 137–146 & 189–298); and magenta, “arm” protrusion (residues 147–188) used for RBP attachment. Red arrows indicate estimated locations of the two CBMs of the phage J-1 “evolved” Dit: CBM1 is proposed to insert after the N-terminal ring domain, and CBM2 within a loop of the galectin-like domain [53]. (**B**) Cartoon representation of the putative CBM1 fold (PDB ID: 2XOM) [55] and the known CBM2 fold (PDB ID: 5LY8) [53]; both are rainbow colored from N-terminus (blue) to C-terminus (red). Shown as white sticks are four residues (Asn45, Trp73, Phe216, and Gln219) that are proposed to form the CWPS binding site of CBM2 [53]. (**C**) The CWPS core repeat unit of *L. casei* BL23 [58].

**Figure 4:**
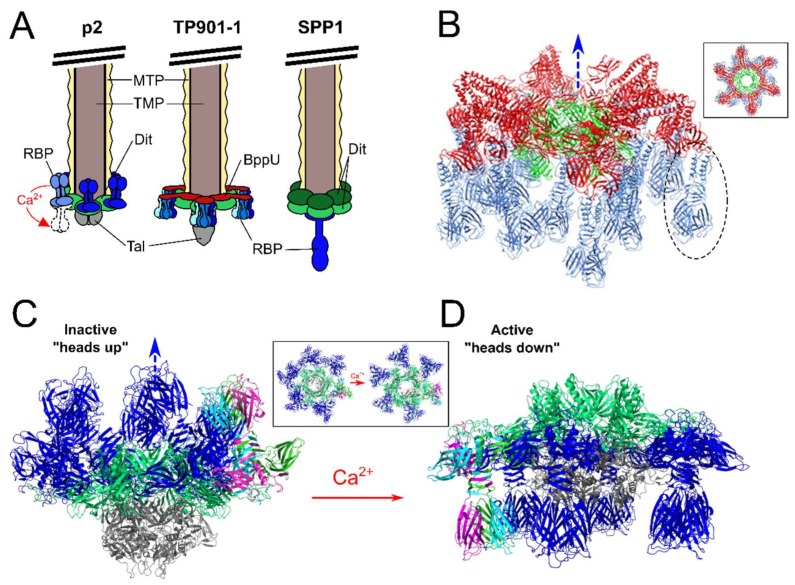
Divergent baseplate architectures of Gram-positive-targeting *Siphoviridae* phages. (**A**) Simplified models of the baseplates from saccharide (pellicle) binding *L. lactis* phages p2 and TP901-1 and protein (YueB) binding *B. subtilis* phage SPP1. Highlighted is the Ca^2+^ activation trigger that induces flipping of RBP trimers to an active state for phage p2. (**B**) Ribbon representation of the TP901-1 baseplate (PDB ID: 4V96 [18]), reproduced with permission from PNAS, Veesler et al. (2012) [18]. The baseplate consists of a central Dit hexamer (green) connected to six copies of trimeric BppU (red). Attached to the end of each BppU are three trimeric RBP complexes (blue), making 54 individual RBP monomers. Missing is the distal Tal complex that would attach to the bottom of the baseplate. A single RBP trimer is highlighted with a dashed circle. Inset box shows the baseplate rotated by 90° around the horizontal axis to provide a view through the tail tube. Compared to the p2 baseplate, there is no conformational changes to the TP901-1 RBP positioning as they are already in an “active state” that is ready for host recognition. (**C**,**D**) Ribbon representations of the p2 baseplate in the inactive state with RBPs in the “heads up” position (PDB ID:2WZP [16]). (**C**), and then switched to the “active” state with RBPs flipped 200° into the “heads down” position (PDB ID: 2X53 [16]) (**D**). The p2 baseplate contains a hexameric ring of Dit (green); trimeric Tal (grey); and six trimeric complexes of RBP (blue). The three chains of a single RBP complex are colored green, magenta, and cyan. Box insets show the baseplate rotated by 90° around the horizontal axis to present a top down view through the tail tube. Blue arrow points along axis towards the phage tail and capsid in (**B**–**D**).

The first gene encountered downstream of the TMP encodes the distal tail protein (Dit). The Dit protein is highly conserved among *Siphoviridae*, targeting Gram-positive [12] and Gram-negative bacteria, such as T5 [51] and lambda [52]. Dit orchestrates the tail and adsorption apparatus assembly, forming a central hexameric hub onto which the distal tail-tube and baseplate components (e.g., Tal and RBPs) are anchored [12]. As an example, the N-terminal domain (residues 1–120) of *Lactococcal* phage p2 Dit forms a central channel ~40 Å wide, while the C-terminal domain (121–298) forms a galectin-like β-sandwich with an “arm” protrusion of ~60 residues that functions as a connector to a single RBP trimer (ORF18) (Figure 3A). Interestingly, the “arm” protrusion is absent from the Dit hubs of phages SPP1 and TP901-1: the SPP1 Dit does not assemble peripheral elements on its baseplate [12] (Figure 3A), whereas the TP901-1 Dit associates with a hexamer of baseplate upper protein (BppU) in a double-disk ring topology with BppU responsible for attachment to the RBPs [18] (Figure 4).

Recently, an “evolved” Dit type has been identified in *Lactobacillus casei* phage J-1 [53] and *Lactobacillus* phage PLE3 [54]. The “evolved” Dit of J-1 features two carbohydrate-binding modules (CBMs) not present in the “classical” Dits observed in phages p2, SPP1, or TP901-1. Whole phage negative stain EM revealed both the J-1 Dit CBMs protrude outwards from the baseplate, unhindered for potential interaction with host receptors [53]. CBM1 (residues 129–322) was identified by HHpred analysis as a structural homolog to a β-1,4-galactotriose CBM (PDB ID: 2XOM) [55], whereas CBM2 (residues 368–614) was solved by X-ray crystallography (PDB ID: 5LY8) to reveal structural similarity to the CBMs found in lectins [53]. A galactose residue was modeled within the binding site of CBM2 revealing numerous conserved features of carbohydrate interactions [56,57], including ring stacking between the galactose and the side chain of Phe216 (Figure 3C). Of the two CBMs, only CBM2 bound to the *L. casei* cells [53]. Although it is incapable of binding to *L. casei* cells when tested as a recombinant protein, the CBM1 may bind in other phage-host systems, or requires incorporation into a complete baseplate structure to become functional. Interestingly, genes encoding RBPs could not be identified within the J-1 genome or other *L. casei* prophages [54], suggesting that the “evolved” Dits have replaced RBPs as coordinators of host receptor specificity for these phages. This coincides with the minimalistic composition of the J-1 baseplate observed by EM, particularly when compared to phages p2 or TP901-1 (Figure 4). Recently, a major cell wall polysaccharide (CWPS) of *L. casei* BL23 was found to contain repeating units of a branched heptasaccharide, rich in rhamnose sugars (Figure 3C) [58]. Pre-incubation of CBM2 with this CWPS or free Rhamnose sugars inhibited binding of phage J-1 to *L. casei* cells, suggesting that this CWPS, and in particular its rhamnose-rich core (2-α-Rha-2-α-Rha-3-α-Rha), is the CBM2 receptor [53].

Host recognition using “evolved” Dits is yet to be determined for other phages, particularly with regard to their location on the phage baseplate. Interestingly, as CBMs are additive instead of substitutive parts of the highly conserved Dit structure [51], similar “evolved” Dits from other phages can be putatively identified based on their larger size (>500 residues) compared to “classical” Dits (~300 residues). As an example, we identified a putative “evolved” Dit (gp16; 522 residues) within the genome of *Listeria* phage A006 [59] (Figure 2). Despite a low sequence identity (15%), HHpred analysis identified a CBM (residues 181–461) with structural similarity to the phage J-1 CBM2 (5LY8; HHpred E-value = 1.5 × 10^−31^), suggesting the Dit complex of phage A006 also plays a role in host recognition in addition to the RBP-encoded downstream (gp17).

## 4. Tail-Associated Lysin (Tal)

After Dit usually follows a tail-associated lysin (Tal)-encoding gene, the product of which assembles into variable sized trimeric complexes on the bottom of the phage baseplate (Figure 4) [60]. Many phage-encoded Tal proteins feature C-terminal peptidoglycan hydrolase (PGH) activity, hence the designation of “lysin” [61]. Although Tal may not play a direct role in host recognition, the degradation of thick peptidoglycan (PG) layers by Tal-associated PGHs ensures more efficient cell wall penetration and phage infection. The enzymatic function of Tal was first described for Tal_2009_ from *L. lactis* phage Tuc2009, which contains a C-terminal M23 peptidase PGH for degrading inter-peptide bridges between the MurNAc subunits of PG [61]. Interestingly, Tal_2009_ and Tal from phage TP901-1 have been shown to undergo proteolytic processing to release their C-terminal PGH domains. This processing does not happen to every phage particle, and thus produces a heterogeneous mix of virions with baseplates containing full-length or truncated Tal complexes [61,62]. It was shown that phages with full-length Tals are more effective at infecting stationary-phase cells, while phages with truncated Tals show higher adsorption efficiencies. Such a heterogeneous population of phages therefore increases the chances of a successful infection against cells where the cell wall composition and level of cross-linkage is unknown [62]. At 919 residues long, the Tal from TP901-1 Tal is longer than its p2 homolog (376 residues). The TP901-1 Tal trimer protrudes from the bottom of the TP901-1 baseplate (Figure 4A), and as described above, features C-terminal PG hydrolase activity that facilitates infection of stationary cells by enabling cleavage of cross-linked PG [61,62]. No hydrolase function could be identified within the p2 Tal or other members of the 936 group. Subsequently, these phages can infect their hosts during the exponential phase, however, they are incapable of infecting stationary cells featuring cross-linked PG [63].

Tal-like structures are not limited to Gram-positive *Siphoviridae*, and are found in Gram-negative *Siphoviridae*, e.g., *Shewanella* prophage MuSo2 (PDB ID: 3CDD), and *Myoviridae*, e.g., phages T4 (gp27; PDB ID: 1K28) [64] and Mu (gp44; PDB ID: 1WRU) [65], as well as the VgrG module that forms the injection apparatus of the bacterial type 6 secretion system (T6SS) [10]. Thus, a common evolutionary origin has been proposed for the baseplate apparatus of tailed phages and bacterial machineries [60].

## 5. Upper Baseplate Protein (BppU)

Large differences between structural proteins encoded downstream of Tal reflect the divergence of structures used for host recognition by different phage baseplates. For instance, phages TP901-1 [18], Tuc2009 [66], and ϕ11 [21] contain an additional baseplate upper protein (BppU) that is not present in the other *Siphoviridae* genomes described in Figure 2. In these phages, BppU functions as a connector between the central Dit hub and the peripheral RBPs. In TP901-1, 18 BppU (gp48) proteins assemble as six asymmetric trimers, forming a ring structure that attaches to the central Dit hub via their N-terminal domains. The C-terminal domains of BppU subsequently attach to three separate trimeric RBP complexes (Figure 4B), providing a total of 54 individual RBPs on the TP901-1 baseplate. Other phages that are proposed to encode similar BppUs are *S. aureus* phage ϕ11 (ORF54) [67] and *C. difficile* phage CDHS1 [5]. Interestingly, for *Listeria* phage A118, BppU appears to be fused to the N-terminus of its RBP (gp20). HHpred analysis identified gp20 as a fusion product of BppU within its N-terminus and receptor binding function within its C-terminus, suggesting that gp20 forms both the attachment to the central Dit hub as well as mediating host recognition [68].

## 6. Receptor Binding Protein (RBP)

The structural composition of the baseplate and RBP morphology varies greatly, depending on the infection process and type of host receptor recognized. Phages using protein-based receptors, such as the *B. subtilis* phage SPP1, typically feature simple baseplate architectures with a single RBP structure. The SPP1 baseplate consists of a central dodecameric organization of two Dit hexamers attached to a single receptor-binding tip formed of a gp21 trimer (Figure 4A) [12]. Host recognition by phage SPP1 occurs in two steps: an initial reversible binding to glucosylated wall teichoic acid (WTA), before rapid and irreversible attachment to an inner membrane protein YueB, a component of the type VII secretion system [14,69]. The interaction with YueB is mediated by the C-terminal region of the gp21 trimer [12,14,15]. The N-terminal domain of gp21 forms a cap within the baseplate that opens up (to allow subsequent DNA ejection) only after gp21 recognizes binds to YueB [14]. Additional rotation of the major tail protein along the entire tail tube assists with DNA ejection [13]. The high affinity and specificity of protein-protein interactions rationalizes the use of a single RBP structure by the SPP1 phage [70]. On the other hand, phages binding saccharidic receptors compensate for the much lower affinity to saccharides (typically within the micromolar Kd range) by increasing the overall number of RBPs—and therefore receptor-binding sites—on their baseplate (Figure 4A). For example, the baseplates of *Lactococcal* phages p2 and TP901-1 contain 18 and 54 RBPs, respectively. Due to avidity, weak affinity binding to a few receptors accumulates to provide sub-nanomolar Kd, and thus irreversible adsorption to the host cell surface.

To date, the majority of Gram-positive targeting RBPs that are structurally resolved are from *Lactococcal Siphoviridae*, specifically phages p2 [71], TP901-1 [72], Tuc2009 [66], 1358 [73], and bIL170 [74], which are described in detail below. Interestingly, despite their low sequence similarity, all of these RBPs form trimeric complexes, with each monomer composed of a conserved modular organization consisting of “head”, “neck”, and “stem” or “shoulder” domains (the latter appear exchangeable); with only phage 1358 RBP being identified as lacking a distinct “neck” domain [73]. From an evolutionary standpoint, the modular RBP organization would allow for efficient exchange of phage head domains (and possibly other RBP components) to produce host range variability. To demonstrate such plasticity, Siponen et al. constructed a functional RBP chimera by fusing the N-terminus and stem domains of phage TP901-1 to the C-terminal head domain of p2 [75]. Structural similarities between the recognition-head domains of the *L. lactis* phage RBPs and those from mammalian Adenoviruses [76] and Reoviruses [77], suggest that despite evolutionarily distant targets and a lack of sequence similarity, these viruses have a common ancestral gene [78].

## 7. Different Baseplates Architectures of the Model *Lactococcal* Phages p2 and TP901-1

Extensive X-ray crystallography and electron microscopy studies of phages p2 and TP901-1 RBPs and baseplates have provided extraordinary levels of detail into their structural organization and activation mechanisms [16,17,18,19,72,79] (Figure 4). Despite strong conservation of their structural genomic modules, these phages present strikingly different mechanisms of host cell attachment. The TP901-1 baseplate appears to be in a constitutively active state, with all 54 putative receptor-binding sites pointing downwards in the direction expected for efficient host adsorption (Figure 4B) [18]. Meanwhile, a recombinant construct of the phage p2 baseplate could be crystallized in two different conformations dependent on the presence of calcium (Figure 4) [16]. When the p2 baseplate was crystallized without calcium, all 18 RBP trimers were positioned in a “heads-up” inactive conformation, facing the tail tube and phage capsid (Figure 4C). Remarkably, when crystallized in the presence of calcium, all the RBPs rotated by 200° to a “heads-down” active position (Figure 4D), with the 18 prospective receptor-binding sites in a downwards position for subsequent host adsorption [16]. In addition, this baseplate rearrangement opens up the trimeric Tal complex to reveal a ~32 angstrom central channel through which dsDNA would be capable of passing [16] (Inset panels, Figure 4C,D). Calcium and other divalent ions are widely used to study and successfully propagate virulent phages [80]. While the presence of calcium ions greatly improves host infectivity of p2-like phages (members of the 936 group), as well as many *Listeria* [40] and *Staphylococcal* [81] phages, the presence of calcium provides no clear advantage to TP901-1-like phages (members of the P335 group) [18]. Coincidently, the calcium binding site observed at the interface of the N-terminal and galectin domains of the p2 Dit [16] is absent in the TP901-1 Dit [18].

Cryo-EM analysis of the *Lactococcal* phage 1358 baseplate revealed a similar organization of its baseplate to that of phage p2, involving the rotation of its RBPs from the “heads up” to “head down” conformations [82]. Interestingly, the presence of calcium was not required to induce this conformational switch [82] suggesting the “active” baseplate conformation occurs concomitantly with the “inactive” conformation, whereas the “active” p2 baseplate conformation appears to be provoked and then stabilized using calcium. The precise role of calcium as well as other molecular activators of baseplate activation and host adsorption requires further investigation. For instance, phage p2 and other 936-group phages have been shown to adsorb to host cells with equivalent efficiency with or without calcium, suggesting that alternative divalent cations (e.g., magnesium or manganese) could replace calcium during phage infection, indicating phage adaptability [80]. However, when calcium is present, the transition through the lytic cycle is expedited for many phages, and provides more efficient production of progeny phages [80]. Nevertheless, the current molecular models of baseplate activation provide a valuable framework for understanding host adsorption mechanisms relevant to other *Siphoviridae*. For example, many of the 1358 phage structural genes share sequence identities with various *Listeria Siphoviridae* [59], suggesting firstly that this uncommon *Lactococcal* phage originated from a *Listeria* phage [83], and secondly that these extraordinary and complex mechanisms of adsorption are likely shared between the saccharide-binding Gram-positive phages.

The following sections summarize our current molecular-level understanding of the interactions between Gram-positive phage RBPs and their diverse array of cell wall receptors.

## 8. Peptidoglycan-Binding Phages

Peptidoglycan (PG) forms the thick mesh-like layers of the Gram-positive cell wall to which other components, including teichoic acids, are covalently attached [84] (Figure 1). PG is a polymer of alternating β-(1,4) linked *N*-acetylglucosamine (GlcNAc) and *N*-acetylmuramic acid (MurNAc) glycan units, cross-linked via peptidyl bridges between MurNAc units of adjacent strands [85]. The mature PG sacculus is typically 15 to 80 nm thick and provides structural rigidity to the cell, which preserves cellular integrity against osmotic forces, while at the same time providing plasticity to allow modification of bacterial shape during various stages of growth, division, and infection [85]. PG has been identified as a receptor used by various phages. For example, *Clostridium botulinum* phage α2 interacts with both GlcNAc and MurNAc residues of PG as its primary receptor [86], and *Bacillus thuringiensis* phage Bam35 binds only MurNAc units of PG [87]. Host adsorption and plaque formation by phage Bam35 could be inhibited by incubating the phage with purified PG (chemically treated to remove proteins and teichoic acid) or a solution of free MurNAc; the addition of free GlcNAc did not affect plaque formation [87]. Due to its ubiquitous presence within all bacterial cell walls, as a single subunit, MurNAc is unlikely to be the sole epitope for phage Bam35 interaction, and it is suggested that phage Bam35 also binds the species’ variable peptidyl bridge between linked MurNAc subunits [87]. For many phages, binding to PG occurs alongside recognition of other cell wall components, e.g., *Listeria* phage A511 [20] and *Staphylococcus aureus* phage ϕ11 [67], where both additionally bind wall teichoic acids. It is therefore proposed that PG plays a synergistic or additive role during host cell interaction by the phage and its RBPs.

## 9. Teichoic Acid-Binding Phages

Teichoic acids are a highly diverse group of glycopolymers composed of phosphodiester-linked polyol repeat units that play critical roles in bacterial adsorption to host cells and the regulation of cell wall autolysins [88]. Teichoic acids are represented as lipoteichoic acids (LTAs), which are anchored to the cell membrane via a diglyceride group, or WTAs, which are covalently linked to the PG using a conserved disaccharide linker [89]. WTAs are the most abundant surface molecule in the cell walls of the *Bacillales* order of Gram-positive bacteria, spanning the genera *Bacillus*, *Listeria*, and *Staphylococcus* [88,90]. For *S. aureus* and *B. subtilis*, it is estimated that every ninth MurNac residue of the PG is linked to a WTA polymer between 40 and 60 polyol repeats [88,89,91]. Due to the extensive decoration of the cell wall by TAs, it is not surprising that a large proportion of phages employ this molecule as a recognition element or receptor (Table 1).

## 10. Lipoteichoic Acid-Binding *Lactobacillus* Phages

LTA plays an important role in host recognition by *Lactobacillus* phages [97]. The RBP of *Lactobacillus* phage LL-H (gp71) is proposed to form complexes between LTA-coordinated Ca^2+^ during its adsorption and subsequent penetration through the *Lactobacillus* PG layer [93]. The level and specific decoration of d-alanine and α-glucose substitutions on the poly-glycerophosphate backbone of LTA plays a critical role in the adsorption efficiency for phage LL-H [98] and other *Lactobacillus* phages [93,99]. Interestingly, mutant LL-H phages with expanded host ranges feature various point mutations within the C-terminal regions of the gp71 RBP, providing additional evidence for gp71 as a determinant of receptor specificity [93]. Such mutant RBPs expanded phage host range to include *L. delbruckii* strains that lack glucose decorated LTA [93], thus suggesting that the mutant phages do not require reversible interactions with the LTA, and can instead attach irreversibly to the host cell; however, this may come with a loss of RBP specificity [93,99].

## 11. Wall Teichoic Acid-Binding *Listeria* Phages

**Figure 5:**
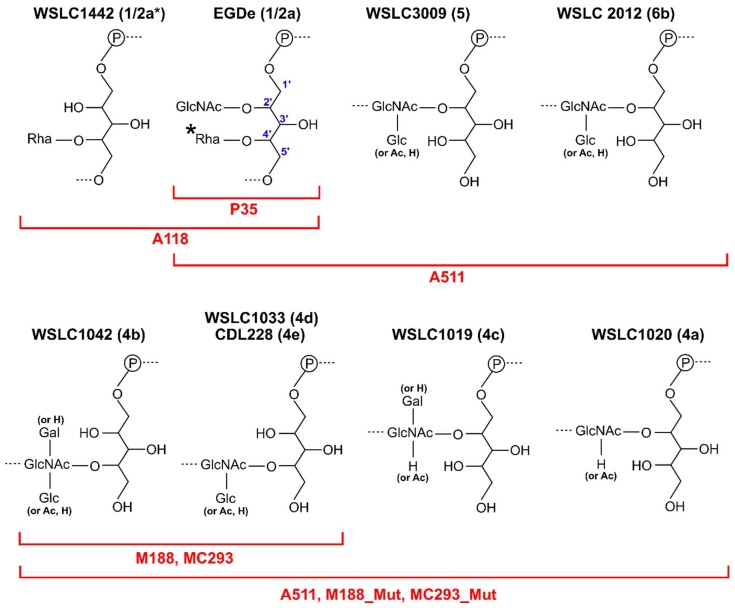
Diversity of WTA structures for *Listeria* serovars produces highly specific receptors. Shown are the repeating units of WTA decorations for *Listeria* serovars 1/2, 4(a–e), 5, and 6. Indicated in red are the host ranges of different *Listeria* phages that adsorb and infect different serovar-specific WTA decorations. * *Listeria* SV 3 strains are devoid of the C4 Rha substitution. WTA structures are all derived from *L. monocytogenes* strains, except for *L. innocua* SV 6b strain WSLC 2012 [20,100]. Abbreviations: GlcNAc, *N*-acetylglucosamine; Rha, Rhamnose; Glc, Glucose; Gal, Galactose; Ac, Acetate; P, phosphate.

At least 12 serovars (SV) have been identified for *L. monocytogenes* based on the variable compositions and substitutions to their WTA ribitol-phosphate (RboP) backbone [100], with SV 1/2a, 1/2b and 4b primarily associated with human infections [101]. *Listeria* phage typically demonstrate a host range preference dependent on SV type [20,40,59,96,100]. For instance, phages A118, A006, and P35 will only adsorb and infect *Listeria* SV 1/2 strains [96] (Figure 5). The SV 1/2 type is defined by substitution of GlcNAc and Rhamnose (Rha) sugars onto the C2 and C4 positions, respectively, of the WTA RboP subunit [100]. Both saccharides play critical roles in host adsorption for various SV 1/2 infecting phages. For instance, the virulent *Siphoviridae* P35 can only adsorb and infect *Listeria* with both GlcNAc- and Rha-decorated WTA, and it is incapable of infecting strains deficient in either saccharide [96], while phage A118 appears to only require Rha-decorated WTA, and has been shown to infect strains that lack GlcNAc within their WTA [96,102]. HHpred analysis identified the RBP of P35 (gp16), which features strong homology within its C-terminal region to a putative prophage tail protein (gp18) encoded in the genome of *L. monocytogenes* EGDe [96]. The cell wall binding range of GFP-tagged P35 RBP correlated directly with the whole P35 phage infection range, as it could only decorate SV1/2 type WTA containing both saccharide substitutions [96]. Two candidate RBPs, gp19 and gp20, were identified within the A118 genome, which also demonstrated strong structural homology to other phage proteins involved in host recognition; for instance, the C-terminus of gp20 is structurally related to the RBP (gp49) of *L. lactis* phage TP901-1 [19]. Both gp19 and gp20 were capable of binding *Listeria* SV 1/2 cells, suggesting both as determinants of host-specificity for phage A118. These RBPs could also bind GlcNAc-deficient WTA, suggesting that the Rha-decorated WTA is a key determinant of binding range. The incubation of phage A118 with antibodies raised against gp19 and gp20 (α-gp19 and α-gp20) or the A118 Tal (α-gp18), completely abolished phage adsorption and infection. Meanwhile, antibodies raised against other components of the tail or baseplate (α-gp16, α-gp17, α-gp21) had no effect on adsorption or infectivity [96]. Both gp19 and gp20 are thus required for host adsorption and are essential components of the baseplate; however, their exact location and precise roles in host recognition are yet to be determined. Combining EM data and HHpred homology predictions, two models of the A118 baseplate have been proposed, which reveal high similarity of A118 to the model *Lactococcal* phage TP901-1 [68,96] (Figure 4B). As observed for TP901-1, the baseplate features a central hexameric ring of Dit (gp17), a trimeric Tal (gp18) complex, and six trimeric RBP complexes (gp20) as peripheral elements [68]. The similarity of A118 and TP901-1 baseplates suggested that phage A118 could have derived from TP901-1 potentially through host species exchange [68]. While the A118 gp20 is proposed as the RBP, the A118 gp19 was identified as a putative esterase, suggesting this protein possibly features enzymatic activity [68], potentially as a cell wall-degrading component of the baseplate to assist host infection. Immuno-electron microscopy revealed gp19 as a part of the upper and lower baseplate; however, it remains uncertain how this extraordinary protein could potential play both roles in host recognition and/or cell wall degradation. Further detailed structural studies on the A118 baseplate will be necessary to realize its unusual host adsorption mechanism.

SV 4b/4e specific phages M188 and MC293 are members of the same cluster of temperate phages as A118, A500, and A006, with high conservation of their tail morphogenesis sub-module [103] (Figure 2). As SV 4b/e specific phages, their RBPs are proposed to require terminal GlcNAc at position C4 of the RhoP backbone for attachment (Figure 5). Continued exposure of both phages to an insensitive SV 4c *L. monocytogenes* strain generated spontaneous mutants, 188_Mut and 293_Mut, with expanded host ranges including SV 4a and 4c strains as well as SV 4b/e strains [103]. Sequence analysis of their baseplate encoding genes revealed extensive mutation within gp19 of phage 118_Mut and gp20 of 293_Mut. For instance, the latter retained only 82% nucleotide sequence identity (49/238 residues mutated) to parental MC293 gp20 [103]. Based on HHpred analysis and relative position within the tail morphogenesis sub-module, M188 gp19 and MC293 gp20 were identified as RBPs with structural homology to the RBP (gp49) of TP901-1 [103]. EM analysis of the MC293 baseplate revealed a six-pointed star confirmation, as observed for the TP901-1 baseplate structure, suggesting a similar ring of six RBP complexes on the periphery of the MC293 baseplate.

Despite the vast majority of characterized *Listeria* phages belonging to the *Siphoviridae* family, a handful of *Myoviridae* and their RBPs have been described; however, no *Listeria*-infecting *Podoviridae* have been isolated, and it is unclear if these even exist [40]. *Myoviridae* A511 and P100 are members of the *Spounavirinae* sub-family, featuring other Gram-positive-targeting *Myoviridae*, i.e., *S. aureus* phage Twort and *Bacillus* phage SPO1 [20,104]. A common feature among *Myoviridae* phages are the six long tail fibers (~150 nm) protruding from the baseplate, which are responsible for host recognition via reversible interaction with host bacteria. While the LTFs have been well-characterized structurally and functionally for Gram-negative-infecting phages [11,105,106,107,108], little is known about the interaction mechanism of LTFs targeting Gram-positive hosts. For instance, while the A511 LTF has been identified (gp104), it has yet to be shown how this protein functions during Gram-positive host recognition. EM analysis of phage A511 has provided a detailed model of its baseplate architecture, providing a representative model of host adsorption for A511 and other *Spounavirinae* members [20]. HHpred and gene cluster analyses have identified a putative RBP (gp108) for phage A511 [20], while EM analysis of phage A511 after α-gp108 gold immuno-labeling identified the protein as a peripheral component of the phage baseplate. GFP-tagged gp108 construct was also able to decorate the same *Listeria* SV 1/2, 4, 5, and 6 cells as infected by the whole A511 phage, providing clear evidence for the role of gp108 as a RBP. Interestingly, all SV types bound by gp108 and infected by the whole phage feature a GlcNAc substitution at positions C2 or C4 of the WTA, suggesting a role for this sugar for host recognition. As expected, gp108 was incapable of binding *Listeria* strains that were deficient in GlcNAc-decorated WTA; for instance, against *Listeria* WSLC 1442 (1/2a*) lacking GlcNAc at position C2 (Figure 5) [109]. Interestingly, gp108 is incapable of binding *Listeria* SV 3 cells, which also contain GlcNAc at position C2, but lack the Rha at position C4. Similarly, the whole phage A511 cannot infect SV3 strains either. It was proposed that Rha is therefore also required for gp108 interaction of the WTA receptor [20]. Gp108 retained the ability to bind SV 4, 5, and 6 strains despite their WTAs being devoid of any Rha decoration; it was thus hypothesized that gp108 interacts with other cell wall components, with the PG as the most likely candidate [20,110].

In conclusion, a variety of *Listeria* phage RBPs have been identified as baseplate-attached determinants of host range, playing essential roles in phage adsorption against various *Listeria* SVs [20]; however, the molecular details of RBP binding to specific WTA substitutions remains to be studied. Unfortunately, there are no high-resolution structures of *Listeria* phage RBPs available. Our current research aims to elucidate such atomic structures and provide the fine details of interactions by various *Listeria Siphoviridae* and *Myoviridae* RBPs to their host receptor(s).

## 12. Wall Teichoic Acid-Binding *Staphylococcus* Phages

Similar to *Listeria* phages, WTA is an essential host receptor used by all known *Staphylococcal* phages [24,111]. Compared to the highly variable WTA compositions observed between *Listeria* SV [100], there appears to be less variability between *Staphylococcal* WTA decorations [24]. *S. aureus* WTAs are composed of a similar RboP backbone featuring three possible substitutions: d-alanine, α-*O*-GlcNAc, or β-*O*-GlcNAc [88]. Interestingly, *S. aureus* strains containing WTAs with only GlcNAc decorations appear to be resistant to infection by all known *S. aureus Siphoviridae* [111] and *Podoviridae* phages [24]. Interestingly, infection by *Podoviridae* Φ44AHJD, Φ66, and ΦP68 requires a precise WTA glycosylation pattern dependent on the stereochemistry of the GlcNAc substitutions [24]. *S. aureus* mutant strains featuring only α-*O*-GlcNAc-decorated WTA are resistant to infection by the above mentioned *Podoviridae* phages; however, *S. aureus* mutants containing only β-*O*-GlcNAc-decorated WTA are susceptible to phage infection [24]. Thus, only the β-isomer of GlcNAc is utilized by these *Podoviridae* as a receptor. Wild type *S. aureus* normally features both α-*O*- and β-*O*-GlcNAc-decorated WTA and is therefore inherently susceptible to phage infection. Interestingly, all tested *Siphoviridae* can infect *S. aureus* regardless of the stereochemistry of the WTA GlcNAc substitutions, and can adsorb and infect mutant strains featuring either α-*O*- or β-*O*-GlcNAc-decorated WTAs with comparable efficiency to that of wild-type cells [67,111]. Remarkably, the GlcNAc substitutions are not even necessary for infection by the *S. aureus Myoviridae* ΦK and Φ812, which only require the RboP backbone for adsorption [111]. However, a secondary receptor is most likely recognized by these phages that has yet to be identified. *Staphylococcal Myoviridae* ϕSA012 contains two RBPs, gp103 and gp105, with the former binding to α-GlcNAc substituted WTA and the latter to the backbone of the WTA [112]. ϕSA012 belongs to the same *Spounavirinae* sub-family, including *Listeria* phages A511 and P100 [20].

**Figure 6:**
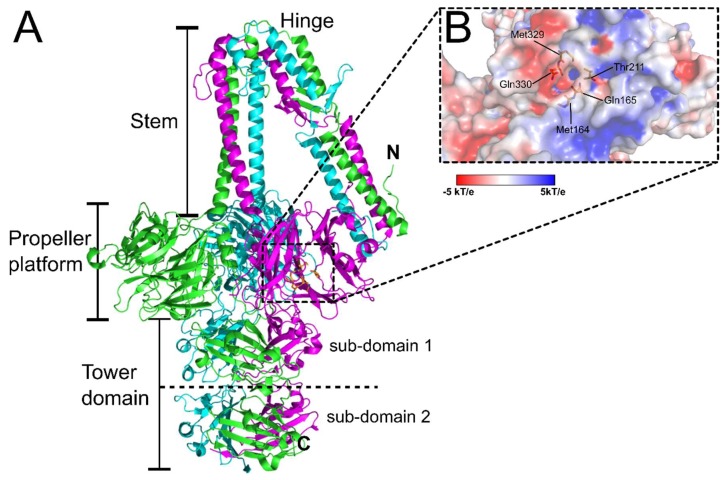
The RBP (gp45) of *Staphylococcal* phage ϕ11. (**A**) Cartoon representation of the trimeric complex of gp45 (PDB ID: 5EFV) [21] with individual chains colored green, magenta, and cyan, and distinct structural domains indicated. Shown as orange sticks and highlighted by the dashed box are the residues lining a putative GlcNAc binding site cavity featured within each propeller platform domain. (**B**) Zoomed in surface representation of the putative GlcNAc binding cavity colored according to electrostatic surface potential (red = negative charged, white = neutral charged, and blue = positive charged (±5 kT/e)), with all cavity forming residues labeled and represented as orange sticks, as previously described [21].

Among the *S. aureus* phages, ϕ11 is one of the best studied *Siphoviridae*, due to its application as an efficient transducer of genetic markers for *S. aureus* strains [67]. As described for other *Siphoviridae*, ϕ11 can adsorb and infect cells with either α- or β-GlcNAc-decorated WTA [67,113]. In addition, a secondary and essential receptor of ϕ11 has been identified as the *O*-acetyl groups at the 6-position of the MurNAc subunits of the PG layer [67]. The crystal structure of the ϕ11 RBP (gp45) (PDB ID: 5EFV) revealed an unusual five-bladed β-propeller domain that contains a putative GlcNAc binding cavity involved in host recognition (Figure 6) [21]. Similar six- and seven-bladed β-propeller domains have been identified in the RBP of the broad host range of *Salmonella* and *E. coli* phage PRD1 (RBP-P2; PDB ID: 1N7U) [114] and the peripheral tail protein gp131C of a *Pseudomonas* phage ϕKZ (PDB ID: 4GBF) [115], respectively. EM reconstruction of the ϕ11 baseplate identified six gp45 RBP trimers assembling around the baseplate in a downwards orientation, similar to TP901-1 (Figure 4B), suggesting the phage exists in a similar constitutively active configuration [21].

## 13. Pellicle-Binding *Lactococcal* Phages

Phages infecting *Lactococcus lactis* strains are predominantly members of the *Siphoviridae* family. Virulent *L. lactis* phages and other phages of lactic acid bacteria can contaminate and lyse important starter cultures used for milk fermentation. The significant economic impact of *L. lactis* phages on the dairy industry has led to them being one of the most studied families of Gram-positive targeting phages [116]. *Lactococcal* phages are classified into 10 genetically distinct groups, with groups 936, P335, and c2 accounting for around 80%, 10%, and 5% of all known phages, respectively [79,116]. Phages p2 and TP901-1, as discussed earlier (Figure 4) are members of group 936 and group P335, respectively. Instead of binding to a saccharide receptor, the c2 group recognizes a cell membrane protein termed “phage infection protein” (PIP) [117,118], which is orthologous to the YueB protein recognized by phage SPP1 [15,119].

**Figure 7:**
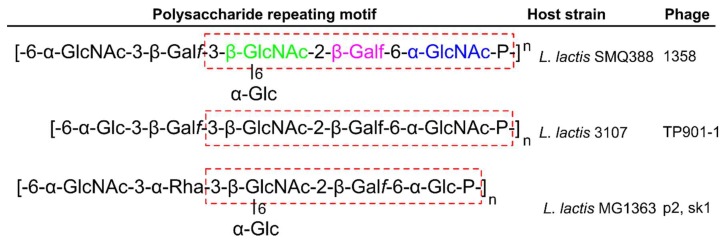
The *Lactococcal* pellicle phage receptor. Shown are the repeating phosphohexasaccharide units of strains MG1363 and SMQ-388 [22,73], and the phosphopentasaccharide unit of strain 3107 [121]. Dotted red box highlights the core trisaccharide for each pellicle; for strain MG1363, colors correspond to Figure 8. Listed are the phages that infect the host strain by targeting these specific pellicle repeats. Abbreviations: GlcNAc, *N*-acetylglucosamine; Galf, Galactofuranose; Glc, Glucose; Rha, Rhamnose; P, phosphate.

The majority of *Lactococcal* cells contain LTA and WTA; however, there are many strains that lack one or the other, suggesting that they play a dispensable role in cell function, or that their roles can be replaced by other cell wall components [99,120]. *L. lactis* cells have been shown to produce a thin polysaccharide pellicle layer on the outer surface of their cells that is suggested as the receptor for *L. lactis* phages [22]. Figure 7 describes the chemical composition of the three currently known pellicle-repeating units, with strains MG1363 and SMQ-388 containing repeating phosphohexasaccharides [22,73], and strain 3107 containing repeating phosphopentasaccharides [121]. A core trisaccharide producing the three known pellicle-repeating units is proposed as the receptor—or a part of the receptor—for *Lactococcal* phages [99].

## 14. Receptor Binding Sites of *Lactococcal* Phage RBPs

**Figure 8:**
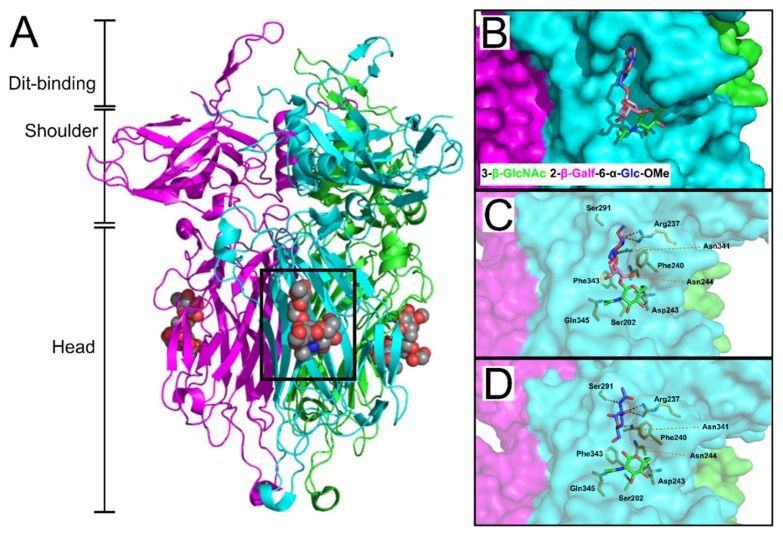
The pellicle core trisaccharide binding site of *L. lactis* phage 1358. (**A**) Cartoon representation of the *L. lactis* phage 1358 RBP (ORF20) (PDB ID: 4RGA) [122] with the synthesized trisaccharide (resembling the native core structure) shown as spheres (carbon = grey, nitrogen = blue, oxygen = red) bound within the same cleft on the side of the head domain of each monomer (colored cyan, green, and magenta). Highlighted are the receptor-binding head domain, central shoulder domain, and the N-terminal Dit-(interaction) domain; the function of the latter domain is to attach the RBP to the central Dit hexamer ring of the baseplate. (**B**) Surface representation of the saccharide binding cleft of the 1358 RBP, with the core trisaccharide shown as sticks. (**C**) The interaction between the trisaccharide to the 1358 RBP binding cleft involves hydrogen/ionic bonding (yellow dashed lines) with various cleft-forming residues. (**D**) Individual GlcNAc residues (blue and green; PDB ID: 4L92) [73] interact with equivalent hydrogen/ionic bonds as their respective single saccharides of the synthetic trisaccharide.

Virulent phage 1358 is the reference member of a rare group of phages infecting *L. lactis* [83]. Recently, McCabe et al. co-crystallized the phage 1358 RBP with a synthetic trisaccharide (TriS) representing the core of the SMQ388 pellicle (3-β-GlcNAc-2-β-Gal*f*-6-α-Glc-OMe) to provide unprecedented molecular details of an RBP-host interaction [122] (Figure 8). TriS was located within a deep crevice in the middle of the RBP head domain, sandwiched between two hydrophobic phenylalanine residues (Phe240 & Phe243) and strongly anchored by hydrogen bonding between the two terminal sugar residues (Figure 8B,C). Farenc et al. (2014) first solved the crystal structure of 1358 RBP in complex with GlcNAc (Figure 8C,D) or glucose 1-phosphate (GlcP) monosaccharides. Remarkably, these single saccharides form equivalent interactions as observed for the Glc-OMe and GlcNAc components of the TriS interaction, respectively [73], confirming the location of the receptor-binding site within the crevice, as well as the important residues involved in receptor binding [122].

**Figure 9:**
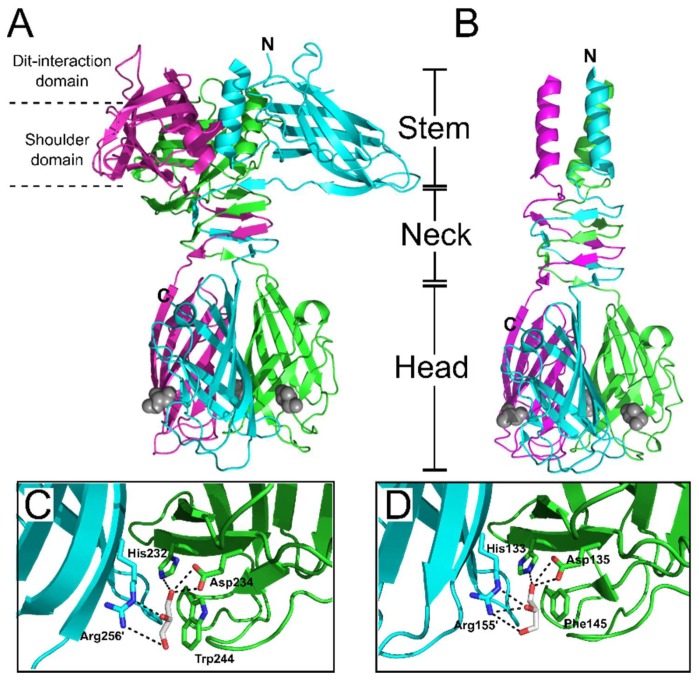
Structures of p2 and TP901-1 trimeric RBPs and putative binding sites. Ribbon representation of the p2 RBP trimer (ORF18) (PDB ID: 1ZRU [71]) (**A**), and TP901-1 RBP trimer (ORF49) (PDB ID: 2F0C [72]) (**B**), with monomers colored green, magenta, and cyan. Both RBPs have distinct stem, neck, and head sub-domains, the functions of which are described in the main text. The two additional sub-domains within the p2 RBP are the Dit-interaction domain and the shoulder domain. In both structures, glycerol molecules, co-crystallized with the RBPs, are shown as grey spheres. (**C**,**D**) Close-up view of glycerol in the receptor-binding site of the RBPs of phages p2 (**C**) and TP901-1 (**D**), with glycerol and side chains of interacting residues shown as sticks.

Similarly, high-resolution crystal structures of the phages p2 [71,78] and TP901-1 [72] RBPs were solved with glycerol molecules bound at each of the three interfaces between their head domains (Figure 9). The glycerol molecules interacted by hydrogen bonding to equivalent side chains: His232, Asp234, and Arg256 for p2 RBP; and His133, Asp135, and Arg155 for TP901-1 RBP (Figure 9C,D). Furthermore, the hydrophobic face of glycerol packs against a tryptophan (Trp244) or phenylalanine (Phe145) side chain in the p2 or TP901-1 RBP, respectively. The common features of the glycerol binding as observed for saccharides [57], suggested that the interfaces between head domains are the receptor binding sites for the TP901-1 and p2 RBPs. This is highly dissimilar to the identified receptor-binding site of phage 1358 RBP, which exists as a cavity on the side of each individual head domain (Figure 8B) with no possible ligand binding observed between the 1358 RBP head domain interfaces. Due to the proximity of glycerol to Trp244 in the p2 RBP, fluorescence quenching experiments measured the affinity of glycerol, galactose, phosphoglycerol, and a MurNAc-dipeptide to this prospective binding site, providing measured Kd values between 0.26 to 0.12 μM [71]. After generating a Phe145Trp mutant of the TP901-1 RBP, fluorescence quenching experiments produced similar affinity constants for glycerol and various saccharidic molecules to those measured for the p2 RBP [72]. Furthermore, surface plasmon resonance (SPR) measurements of the interaction between the p2 trimeric RBP head domain to purified host *L. lactis* MG1363 pellicle demonstrated a strong interaction, also within the nanomolar range (Kd = 230 ± 40 nM) [17]. Although this interaction was weaker than protein receptor-binding phages, for instance phage PRD1 binds to its host receptor with a Kd of 0.2 nM [123], the weaker interaction is compensated by the high-avidity provided by the 18 potential receptor binding sites distributed between the six RBP trimeric complexes present on the p2 baseplate [17]. For a more comprehensive overview of *Lactococcal* phages and their host recognition mechanisms, we highly recommend reading the textbook “Gram-positive phages: From isolation to application” edited by Mahony and Van Sinderen (2015) [124], as well as a recent review by Mahony et al. (2017) [99].

## 15. Flagellotropic Phages

Larger bacterial appendages, such as the flagella and pili, can also be used as phage receptors [23,125,126,127,128]. Due to the low probability of a phage encountering suitable host cells in certain environments, the recognition of motile flagella—that can protrude up to 15 μm from the bacterial body—greatly improves the possibility of a phage interacting with a suitable host [128]. Such flagellotropic phages have been described for both Gram-negative [125,126,128,129] and Gram-positive bacteria, i.e., phages PBS1 [23,127] and PBP1 [127,130] that infect *Bacillus subtilis* and *Bacillus pumilis*, respectively. After reversible attachment to the flagella, counter-clockwise rotation of the flagella filament apparently moves the phage particle along the flagella to the cell body, where secondary receptors are irreversibly bound, eventually triggering DNA ejection into the host cell. The flagella-binding RBP has been identified on most flagellotropic phages as a single, long RBP protruding from the bottom of the baseplate [23,126,130]; however, capsid-bound RBPs have also been identified [128]. The unusual mechanism of flagella attachment has also been observed using EM for PBS1 [23] and PBP1 [130]. When flagella are removed, truncated, or damaged (either by mutation or mechanical force) the ability of phages to bind the host cells is significantly reduced [23,128,130], and is proposed to ensures the phage ultimately injects its genome into a metabolically active bacterium, and thus the required environment for phage progeny production [128].

## 16. Future Perspective

Studying phage-host interactions on a molecular level is essential for accelerating the exploitation of phages for remediative and diagnostic purposes. Phage host range can vary greatly; as some phages infect single strains, others are capable of infecting across bacterial species or genera. A highly specific host range is advantageous for applications that requirie a high specificity of detection or killing of a certain strain; however, it can also be detrimental when a phage is used with the intention to eradicate pathogenic bacteria. For instance, the therapeutic use of phages (phage therapy) is a desirable alternative or supplemental treatment together with antibiotics against bacterial infections. A major bottleneck of phage therapy is the limited host range of phages. A potential solution is phage genome engineering. By rational design, virulent phages can be armed with additional antimicrobial properties or engineered with broader host ranges by adapting their RBP binding targets [32,131,132,133,134]. The development of synthetic phage genomes could allow the engineering of phages with adapted or defined host specificities against select bacterial species [134]. Ando et al. used phage genome assembly in yeast to generate synthetic phage genomes [134]. They managed to switch host specificities in phages targeting different bacterial species; however, their method requires highly competent cells and is largely limited to phages infecting Gram negatives. Recently, an alternative method for phages infecting Gram positives has been reported, based on rebooting of phage genomes in bacterial L-form cells [32]. This method uses cell wall-deficient *Listeria* that can be transformed with full length in vitro assembled phage genomes. Moreover, *Listeria* L-forms are also capable of cross-genus rebooting of phage genomes from not only *Listeria*, but also in *Staphylococcus* and *Bacillus*. In conclusion, these methods could be used to generate whole RBP libraries to screen for more effective broad host range phages, with the potential to reduce the need for phage cocktails in medical application and develop standardized drug treatments with easier regulatory approval. However, understanding the fine details of host receptor interaction is imperative to allow a rational, structure-guided approach towards RBP modification.

## Figures and Tables

**Table 1:** List of Gram-positive-targeting phages and confirmed host cell receptors.

Phage	Family	Main Host	Receptor(s)	Receptor Details	RBP Known (?)	References
γ	*Siphoviridae*	*Bacillus anthracis*	cell-wall-anchored protein	gamma phage receptor (GamR)	-	[92]
SPP1	*Siphoviridae*	*Bacillus subtilis*	wall teichoic acid (WTA) and membrane protein	Reversible binding: GlcNAc substituted poly(glycerophosphate) of WTA. Irreversible binding: membrane protein YueB	ORF21	[14]
PBS1	*Siphoviridae*	*Bacillus subtilis*	Flagella	Reversible binding to flagella filament (only to motile cells)	-	[23]
LL-H	*Siphoviridae*	*Lactoccus delbrueckii*	LTA	Reversible binding: Glucose substituted LTA. Irreversible binding: negatively charged glycerol phosphate groups of LTA	SP58 (*g71*)	[93]
c2		*Lactoccus lactis*	membrane protein and PG	Reversible binding: Rhamnose residues of PG. Irreversible binding: membrane “phage infection protein” (PIP)	ORFl10	[94,95]
p2	*Siphoviridae*	*Lactoccus lactis*	Pellicle	phosphohexasaccharide pellicle	ORF18	[17]
TP901-1	*Siphoviridae*	*Lactoccus lactis*	Pellicle	phosphopentasaccharide pellicle	ORF49	[18]
A118	*Siphoviridae*	*Listeria monocytogenes*	WTA	GlcNAc substituted WTA	ORF20 and ORF19	[96]
P35	*Siphoviridae*	*Listeria monocytogenes*	WTA	GlcNAc and Rhamnose substituted WTA	ORF17	[96]
A511	*Myoviridae*	*Listeria monocytogenes*	WTA and PG	WTA and peptidoglycan	ORF108	[20]
ϕ66	*Podoviridae*	*Staphylococcus aureus*	WTA	β-*O*-GlcNAc substituted WTA	-	[24]
ϕ11	*Siphoviridae*	*Staphylococcus aureus*	WTA and PG	α- or β-GlcNAc substituted WTA and 6-*O*-acetylated MurNAc of PG	ORF45 and ORF50 (tail fiber)	[21,67]
